# Responding to excessive alcohol consumption in third-level (REACT): a study protocol

**DOI:** 10.1186/s12913-018-3173-z

**Published:** 2018-05-11

**Authors:** Martin P. Davoren, Susan Calnan, Judith Mulcahy, Emily Lynch, Ivan J. Perry, Michael Byrne

**Affiliations:** 1 Sexual Health Centre, 16 Peters Street, Cork, Ireland; 2 REACT Project, Student Health Department, University College Cork, Ardpatrick, College Road, Cork, Ireland; 3 School of Public Health, University College Cork, 4th Floor Western Gateway Building, Western Road, Cork, Ireland

**Keywords:** Students, Alcohol, Intervention, University, Ireland, Multi-component, Complex

## Abstract

**Background:**

Problem alcohol use is an ongoing, worldwide phenomenon of considerable concern. Throughout the past 20 years, national policies have noted the importance of students when tackling alcohol consumption. Considering alcohol is a multifaceted issue, a multi-component response is required to combat its excessive use. This protocol sets out the approach used for developing, implementing and evaluating the REACT (Responding to Excessive Alcohol Consumption in Third-level) Programme.

**Methods/design:**

This evaluation will provide the evidence base for programme development, implementation and improvement. Stage one involved defining the multi-component intervention. This was developed following a systematic review of existing literature and a Delphi-consensus workshop involving university students, staff and relevant stakeholders. Following this, the programme is being implemented across the Higher Education sector in Ireland. A number of Higher Education Institutes have declined the invitation to participate in the programme. These institutions will act as control sites. Each intervention site will have a steering committee whose membership will include a mix of students and academic and student service staff. This steering committee will report to the REACT research team on the implementation of mandatory and optional action points at local sites. An online cross-sectional study at baseline and two-years post intervention will be utilised to determine the impact of the REACT programme. The impact assessment will focus on (1) whether the intervention has reduced alcohol consumption among third-level students (2); whether the programme altered students attitudes toward alcohol and (3) whether the programme has decreased the second-hand effects associated with excessive consumption. Finally, qualitative research will focus on factors influencing the take-up and implementation of this programme as well as students’ views on the initiative.

**Discussion:**

Alcohol consumption has remained on the policy agenda at both national and international level over recent decades. Students are regularly among the highest alcohol consumers, yet university management and public policymakers struggle to tackle this burgeoning issue. The REACT Programme provides a structure to translate policy into practice for those seeking to reduce hazardous alcohol consumption and related harms among third-level students.

## Background

Problem alcohol use remains a pertinent public health issue [1–4]. In particular, Ireland reports significantly higher alcohol use than its European and worldwide counterparts [5–7]. A recent report highlighted that over half of Irish adults were hazardous alcohol consumers (HAC) [8], defined as a “pattern of alcohol consumption that increases the risk of harmful consequences for the user or others”[8].

Among university students, in a twenty-one country comparison, Irish students reported the highest levels of binge drinking among their university peers [9]. Excessive alcohol consumption is noted as the number one public health concern among university students [10]. In Ireland, hazardous drinking has been identified as the most prevalent substance use problem during university life [11–14]. Recent Irish research noted that two-thirds of students report hazardous alcohol consumption [15, 16]. In addition, this research highlights the burden of related adverse consequences including harmed friendships, relationships, verbal abuse, assault and antisocial behaviour among students as well as the narrowing of the gender gap in alcohol consumption [15, 17].

### National policy

National policies and various reports have noted the importance of students when tackling alcohol consumption [18–22]. The most recent report noted that previous policy reviews have all “recommended the establishment of an integrated national alcohol policy based upon public health principles”[23]. In addition to national policy, the university setting has implemented a range of localised policy and evidence-based interventions to tackle excessive alcohol use [24, 25]. However, a lack of clear evidence is noted. Alcohol consumption is impacted by individual, social and material factors [26]. Thus, responding to excessive alcohol use requires a multi-component approach.

### REACT – Responding to Excessive Alcohol Consumption in Third-level

There is overwhelming evidence that changing people’s health-related behaviour can have a “major impact on some of the largest causes of mortality and morbidity”[27]. Following internal review, the national Health Service Executive (HSE) in Ireland commissioned the research team to develop a public health intervention which would target excessive alcohol consumption in the third-level student population. Considering alcohol is a multifaceted issue, a multi-component response was developed. It was entitled REACT – Responding to Excessive Alcohol Consumption in Third-level. This intervention is informed by international best practice. The project seeks to establish a specially tailored accreditation and award system for third-level institutions (colleges/universities/institutes of technology) that make significant changes within their campuses to tackle the growing issue of excessive alcohol consumption among students. The initiative incorporates a suite of both mandatory and optional action points. Although this method has been widely supported, no clear evidence base has been described. The national HSE continues to support the research team to investigate the evidence base underpinning this approach through implementation and evaluation. The primary aim of this research is to investigate the effectiveness of implementing the REACT project in reducing alcohol consumption and related harm in the third-level student population. Qualitative research on implementation of this intervention as well as students’ views on the initiative will also be undertaken. This paper will outline the stages, methods and data being used to investigate the impact and processes involved in the REACT project.

## Methods/design

### Stage 1: Intervention development

#### Identifying the evidence

In January and February 2015, a literature review focusing on community, school, college and university environmental interventions tackling excessive alcohol consumption was undertaken. Following consultation with a librarian, the search terms used were: alcohol*, student, plan, dashboard, KPI, key performance indicator and plan. Title searches for relevant articles were completed by one reviewer who excluded all irrelevant articles. Results sections of the remaining articles were analysed to investigate suitability. All relevant action plans were extracted and compiled. In total, 918 actions were compiled from relevant articles. All available action points were considered for potential applicability in the Irish third-level setting. This review resulted in 218 available action points. Subsequent reviews eliminated duplication and merged action points. In consultation with the REACT Working Group, 18 mandatory and 34 optional action points were created. This list was considered at a *Knowledge Exchange Forum*. The list of optional action points was divided into four categories based on the National Substance Misuse Strategy in Ireland [12].

### Delphi consensus

#### Participants

In March 2015, a range of senior academic, administrative or professional practitioner staff alongside student representatives from each of the third-level institutions in the Republic of Ireland were invited to attend a *Knowledge Exchange Forum*. In total, there were 46 representatives from 27 organisations in attendance. Each attendee received the Knowledge Exchange Forum booklet one week before the event. The booklet included: (i) an overview of the Delphi consensus method; and (ii) the provisional list of mandatory and optional action points for consideration at the Forum [28]. Participants were asked to rank each action point from what they believed to be the most to least important. Furthermore, individuals were asked to consider the potential impact and applicability in their individual institutions.

At the forum, individuals were randomly assigned seats at 8 tables of 5 to 6 participants. This facilitated the diffusion of institutions and opinion across the forum. Table discussions were recorded. Trained facilitators were responsible for ensuring that each participant shared their views on all action points with the group in an agreed time (30 s per participant per action point). Once each individual had spoken, the facilitator provided the group with the average per table and asked participants to take some time to reflect on the opinion of others. Participants were then asked to re-rank the action points.

### Content analysis and expert consultation

Individual participant scores were entered into Excel. Following analysis, minimum and maximum as well as median scores were computed. Table discussions were transcribed and content analysis was undertaken. Content analysis “is a research method for making replicable and valid inferences from data to their context, with the purpose of providing knowledge, new insights, a representation of facts and a practical guide to action”[29]. This facilitated a relevant description of participant opinion, which supported median scoring. Analysis was conducted using NVivo.

Once analysed, an *Expert Consultation Exercise* was conducted involving the project team, an international alcohol researcher and a student lead. Expert consultation is utilised to gain relevant, informed information when making important decisions. Each action point was reviewed in conjunction with the results of the *Knowledge Exchange Forum*. This meeting yielded the final list of mandatory and optional action points for implementation in the university setting (Tables 1 and 2).

**Table 1:** Final list of mandatory action points included in the REACT Programme

	ACTION POINT	DESCRIPTION
1.	*All incoming students are strongly encouraged to take an online brief intervention tool*	*A target of 33% of incoming first-year students to have completed e-PUB (or other brief intervention tool if already in place) must be met before a college/university/institute of technology is deemed to have achieved this mandatory action point. Statistics should be presented to a relevant college committee on an annual basis*
2.	A college alcohol policy is developed in line with the ‘National Framework to Develop a College Alcohol Policy’	*Develop a college alcohol policy in line with the ‘National Framework to Develop a College Alcohol Policy’*
3.	*President of the college commits to the REACT programme*	*Ensure that the President of the college (or equivalent management figure) signs a three-year commitment to the college actively pursuing the criteria set out by the REACT programme’s Action Point List*
4.	*A Steering Committee is formed, comprising staff and students, and chaired by a senior college official, with meetings taking place twice a year (minimum) and the Action Plan reviewed annually*	*Form a Steering Committee which will:* a) *Have student and staff representation* b) *Be chaired by a senior college official* c) *Have a member of the Gardaí, a member of the local council and a member of the Local Drugs and Alcohol Task Force as committee members* d) *Meet a* minimum *of twice a year* e) *Review the college Alcohol Action Plan* *annually*
5.	*Safety issues in the context of alcohol are considered while planning all large-scale student events*	*Present and discuss an agenda item relating to alcohol and safety issues on the agenda of all SU, Student Society and Student Club meetings regarding large-scale student entertainment events at which alcohol will be available – for example, college balls, gigs and Raise & Give week*
6.	*A tracking and reporting mechanism is established for key alcohol-related harm indicators*	*Establish a tracking and reporting mechanism that will track key alcohol-related harm indicators – for example,* injuries, anti-social behaviour, harm to relationships, studies
7.	The college completes its own evaluation of the effectiveness of the alcohol action plan every three years	*Devise and complete an evaluation strategy to monitor the effectiveness of the alcohol action plan every three years*
8.	*Relevant staff are trained in brief intervention*	*Ensure that key individuals in student health and student experience are able to deliver brief intervention therapy around alcohol misuse and have a clear understanding of the internal referral pathways*

**Table 2:** Final list of optional action points included in the REACT Programme

	ACTION POINT	DESCRIPTION
1.	A specific college official is designated with overall responsibility for the REACT project	*Designate a specific college official to have overall responsibility for the colleges’ REACT programme*
2.	A calendar of events is developed in conjunction with local Students’ Unions	*Develop a calendar of events in conjunction with local Students’ Unions which requires proactive planning*
3.	A reporting mechanism is developed to track high-risk promotions by local licensees	*Develop a reporting mechanism for tracking high-risk promotions by local licensees*
4.	The REACT Training Toolkit is used at class rep training to provide reps with relevant safety information	*Use the REACT Training Toolkit (available* via *the WebApp) for a class rep training session annually with a special emphasis placed on safety* *Invite to the training members of clubs and societies for whom this would hold relevance in event planning*
5.	An alcohol counselling service is available to students	*Provide an alcohol counselling service to the student body*
6.	*A meeting with local stakeholders is held annually*	*Hold a minimum of one meeting annually with local stakeholders (for example, local Gardaí, local residents, local businesses) as a forum to discuss grievances and make suggestions related to students engaged in excessive alcohol consumption*
7.	A visible and accessible referral pathway to a range of internal and external alcohol support services for students is developed	*Develop a visible and accessible referral pathway to a range of internal and external alcohol support services for students* *Include and promote a self-referral route for students as part of this pathway* *Offer training and information relating to the pathway to frontline staff of the college every two years*
8.	Alcohol-free accommodation and alcohol-free social spaces are provided	*Provide alcohol-free accommodation and alcohol-free social spaces*
9.	Partnerships are developed with relevant local community groups	*Develop partnerships with relevant local community groups (for example, local councils, healthy cities committees)*
10.	Late-night transport to student accommodation is provided	*Provide late-night transport to student accommodation for college events/nights out*
11.	A Student Community Support system is developed and implemented	*Develop and implement a Student Community Support system for key student weeks (such as Raise & Give Week and Freshers’ Week)*
12.	Space for Alcoholics Anonymous is allocated	*Make contact with and allocate space for Alcoholics Anonymous to hold meetings for college students*
13.	Local licensed premises are mapped	*Map and update (every two years) all local licensed premises*
14.	*Training on Responsible Serving of Alcohol (RSA) is required for all campus bar staff*	*Require RSA training for all campus bar staff*
15.	The Alcohol Use Disorders Identification Test (AUDIT) is used as the preferred measure of drinking patterns and alcohol-related harm	*Use the AUDIT scale when measuring drinking patterns and alcohol-related harm in health research projects focused on students*
16.	Robust alcohol-related qualitative research is conducted with students	Conduct a high-level, alcohol-related qualitative research* project with students
17.	A PhD/academic researcher is given access to conduct a study on the Action Plan	*Enable a PhD/academic researcher to conduct a study on the effectiveness of the interventions within the Action Plan*
18.	All of the relevant college data related to the Action Plan is provided to the National REACT co-ordinator/researcher	*Provide all of the relevant college data related to the Action Plan to the National REACT co-ordinator/researcher for inclusion in national research*

### Stage 2: Implementation

#### Implementation framework

This intervention will be implemented and evaluated using the MRC framework guidelines on developing and evaluating complex intervention. Complex interventions are usually described as interventions that contain several interacting components, as is the case with the REACT programme [30]. The MRC guidelines, first published in 2000 and further updated in 2006, [31, 32] seek to help researchers and research funders to recognise and adopt appropriate methods. The guidelines outlined in this framework will be used to inform the REACT programme – from its development to implementation.

The MRC framework describes the process of developing and evaluating a complex intervention in terms of several phases, although they may not follow a linear sequence. These phases are: developing an intervention, piloting and feasibility, evaluating the intervention, and implementation. This paper describes in more detail the phases of development and evaluation of the intervention.

#### Implementation

The REACT programme forms part of the wider Health Promoting University ethos, which is based on a ‘settings approach’ to health promotion, whereby the strategic focus is on the whole community and its population, policies and environments rather than solely individuals and problem health behaviours [33]. The concept of the Health Promoting University “means much more than health education for students and staff – it means integrating health into the culture, processes and policies of the university” [33].

In terms of type of intervention, a defining feature of the REACT programme is that it is an environmental rather than educational initiative. DeJong et al. [34] outline that “the chief lesson from work in public health is that people’s behaviour is shaped by their environment, so if we are to change their behaviour we need to change that environment”. The environmental management approach “is intellectually grounded in the field of public health, which emphasises the broader physical, social, cultural and institutional forces that contribute to problems of human health” [34].

The intervention is being implemented across the Higher Education sector in Ireland. A number of Higher Education Institutes have formally declined to participate in the programme. However, some of these institutions agreed to take part in the research either to outline reasons for non-participation or to act as a control site. Each intervention site will have a steering committee (i.e. Steering Committee, mandatory action point 4) whose membership will include a mix of students and academic and student service staff. This steering committee will report to the REACT research team on the implementation of mandatory and optional action points at local sites.

### Stage 3: Evaluation

#### Outcome evaluation

Outcome evaluation measures programme effects in the target population by assessing progress in the outcomes being addressed by the programme. Davies and Macdowall [35] explain that the focus of outcome evaluations tends to be on the behavioural impact of an evaluation, addressing questions such as whether there has been a change in behaviour and what proportion of the target group has heard of the health promotion activities. In outcome evaluations, it is important to distinguish between intermediate and final outcomes – an intermediate outcome, for example, might be a change in attitudes to alcohol use, whereas a longer-term, more final outcome could be an actual reduction in hazardous alcohol consumption levels.

An online cross-sectional study at baseline and at two-year follow-up will be utilised to determine the impact of the REACT programme. A cross-sectional survey was chosen due to the transient nature of the university student environment and the noted decrease in consumption across year of study [15]. It will focus primarily on whether the intervention has succeeded in reducing alcohol consumption among third-level students; and secondly, whether it has increased awareness of the risks of excessive alcohol consumption. It will also aim to gauge students’ awareness of the programme measures and, indeed, if they are aware of the initiative’s existence in the first place. Finally, it will focus on whether the programme impacted student attitudes towards alcohol or decreased the second-hand effects associated with excessive consumption. Collection of baseline data was conducted prior to study implementation. Follow-up will be undertaken two years post intervention.

#### Method

The study will incorporate the AUDIT (Alcohol Use Disorders Identification Test) questionnaire to assess alcohol consumption levels before and after the intervention. The AUDIT was developed by World Health Organization (WHO) as a simple method of screening for excessive drinking [36]. The test seeks to screen for excessive drinking and in particular to help practitioners identify people who would benefit from reducing or ceasing drinking. Attitude and second-hand effect questions will be taken from previous national and international research. Information on associated risk-taking behaviour including smoking, illicit drug use and risky sexual behaviour will also be recorded [14, 37, 38]. All of these instruments have previously shown reliability and validity among a student population [3, 39].

#### Sample

As REACT is a cross-college/university/institute of technology initiative, sampling students across a range of third-level institutions is essential. In total, six intervention institutions and one control institution agreed to participate. Students were sampled using proportional to size sampling using data obtained from the Higher Education Authority (HEA) in Ireland on class sizes per institution for the last academic year. A sample size calculation of 2160 students across six institutions at a 5% margin of error, 95% confidence interval and 50% response distribution was determined.

#### Implementation study

Research on the implementation of a programme or intervention is crucial to help understand why it was or was not successful, looking “inside the so-called black box to see what happened in the programme and how that could affect programme impact and outcomes” [40]. This qualitative study will focus on factors influencing the take-up and implementation of this programme [35]. This will include identifying the barriers and facilitators to implementing the programme, including why some institutions chose not to partake in the programme and why others did. The study will provide important feedback that will be used to inform future programme development and implementation.

#### Method

Steering Committees of each participating institution will be invited to participate in a focus group on the topic of institution implementation. Interviews will also be conducted with the REACT project team. In addition, non-participating institution representatives will be invited to participate in a phone interview to determine their barriers to involvement so that the process evaluation can also identify reasons for non-participation.

Interviews and focus group discussions will be transcribed and analysed to identify key themes. It is proposed that Rogers’ Diffusion Theory will provide the theoretical lens through which to interpret the study findings [41]. In this context, the REACT programme is deemed an innovation, owing to the newness of the initiative (the first programme of its kind to be implemented in Ireland), whose diffusion may be influenced by some or many of the factors outlined in Rogers’ Diffusion Theory.

#### Student feedback study

In addition to research on programme implementation, a qualitative study exploring students’ views on the programme itself will also be undertaken. A key part of any evaluation is obtaining feedback from those who are being targeted by an initiative or intervention. In the case of a programme specifically targeting young people, “feedback from young people is imperative. Evaluation provides young people with an opportunity to comment on how useful they found [the intervention] and this information can then be used for future planning and development” [42] It is intended that this research will contribute to future development and implementation of the REACT programme. It will also help to gauge student attitudes to the issue of alcohol and substance misuse more generally at this significant juncture.

#### Method

Focus groups will be conducted with students at a number of participating institutions (one large city university and one smaller Institute of Technology (IT) located in an Irish town). The focus groups will target a number of key groups identified in the previous quantitative and qualitative research – that is, younger undergraduates, mature students, international students and students who are members of a club or society – owing to the different alcohol consumption levels and patterns found among these groups in the earlier research undertaken.

## Discussion

Alcohol consumption has remained on the policy agenda at both national and international level throughout the past number of decades [23]. Students are regularly among the highest alcohol consumers, yet university management and public policymakers alike struggle to tackle this burgeoning issue. Current levels of student alcohol consumption remain untenable [15, 17]. The university setting lends itself to implementing interventions. As noted previously, while there is considerable evidence internationally on multifaceted action plans aimed at tackling third-level alcohol consumption, there is a dearth of research on how these action plans are developed. The current research seeks to bridge this knowledge gap, detailing how a systematic review of current evidence, followed by a Knowledge Exchange Forum to appraise the suitability of proposed action points, resulted in the final suite of agreed measures. The research also outlines how the programme of measures is to be subsequently implemented and evaluated.

### Policy

Previous research has focused on defining and describing the harmful consumption patterns and second-hand effects of hazardous alcohol consumption among university students [15, 17]. However, few intervention and evaluation endeavours are noted. The REACT project will provide third-level institution management with the evidence base required to tackle excessive alcohol consumption among students.

The Individual, Social and Material (ISM) model contextualises this public health issue in a complex world of rules, norms, regulations, meanings and beliefs [26]. In terms of its theoretical basis, the ISM model draws from behavioural economics, social psychology and sociology. The main aim of this model to make it easier for policymakers and practitioners when tackling complex policy problems where a multi-disciplinary approach is required. The three contexts of ISM can be understood as follows: the ‘individual’ context includes the factors pertaining to the individual that affect the choices and behaviours he or she undertakes; the ‘social’ context includes the factors that exist beyond the individual in the social realm, yet which shape his or her behaviours; the ‘material’ context includes the factors that are ‘out there’ in the wider world [43].

### Individual

Tackling habits, values, beliefs and attitudes surrounding alcohol consumption begins at the individual level. The current plan signals the requirement for brief intervention and alcohol counselling to tackle individual behaviour. Recent research has signalled the efficacy of a range of alcohol interventions. Anderson [44] and Babor [45] have highlighted the beneficial impacts of implementing brief intervention therapy and counselling services to provide individuals with the skill to implement change. Furthermore, online brief interventions have shown a reduction in alcohol-related harm across the student population [46–48].

### Social

The results of this process highlight, first and foremost, the importance of putting in place a structure for change and commitment when creating an alcohol action plan. Organisational commitment is seen as key to behaviour change. Once citizens observe leadership providing commitment, they are more likely to adhere to and work towards a changing environment [49]. The current research follows this framework, appointing the President of each institution as signatory for involvement and prioritising the implementation of a comprehensive alcohol policy as the first steps in setting out a vision for change. In addition, safety issues and tracking of alcohol harm indicators will provide information on norms, roles and identities while ensuring the institution takes a stance on alcohol-related harm. Furthermore, providing class representatives with relevant safety training can motivate advocates among these opinion leaders.

### Material

Finally, this action plan is supported by a range of rules and regulations embedded into the university structure. The development of a college alcohol policy, a steering committee and late night transport result in harm minimisation. This policy outlines the “rules and regulations” [26] to which all individuals within the university setting must adhere. This policy can control for marketing, availability, sports sponsorship and advertising within the confines of the institution’s community [50]. Furthermore, it can provide an advocacy role, taking an institutional stand on a national issue [50]. Recent research noted that students’ perception of policy enforcement resulted in a reduction in alcohol consumption and related harm [51]. The current action plan ensures that each institution will develop, implement and adhere to an evidence-based policy formed on the principles outlined under the National Framework [20].

#### Strengths and weaknesses

The development of the action plan followed core beliefs, knowledge, frameworks and strategies developed at a national level [52]. In addition, the action points resulted from a rigorous systematic review of national and international action plans. Cross-institution collaboration was achieved through discussion and networking at the Knowledge Exchange Forum. Furthermore, clarification of thought through submission to evidence was garnered during expert consultation with an international expert.

Student services, advocates and university management have struggled to tackle the increasing levels of alcohol consumption and related harm among university students. Recently, the Okanagan Charter for Health Promoting Universities and Colleges called for the embedding of “health into all aspects of campus culture, across the administration, operations and academic mandates”. The current paper outlines an evidence-based approach to tackling this burgeoning public health issue across the university system. Currently, the HSE in Ireland is supporting the implementation and evaluation of REACT across third-level institutions. The REACT Programme provides a structure to translate policy into practice and to tackle hazardous alcohol consumption and related harms among third-level students.
